# Metabolic and Hormonal Alterations with Diacylglycerol and Low Glycemic Index Starch during Canine Weight Loss

**DOI:** 10.5402/2012/750593

**Published:** 2012-12-19

**Authors:** Yuka Mitsuhashi, Daisuke Nagaoka, Karen E. Bigley, Tomoshige Umeda, Kazuya Otsuji, John E. Bauer

**Affiliations:** ^1^Companion Animal Nutrition Laboratory, Department of Small Animal Clinical Sciences, College of Veterinary Medicine, Texas A&M University, College Station, TX 77843, USA; ^2^Intercollegiate Faculty of Nutrition, Texas A&M University, College Station, TX 77843, USA; ^3^The Nutro Company, 1550 West McEwen Drive, Franklin, TN 37067, USA; ^4^Minato-Yokohama Animal Medical Research Center, Yokohama, Kanagawa 235-0023, Japan; ^5^Kao Corporation, 2606 Akabane, Ichikai-machi, Haga-gun, Tochigi 321-3497, Japan

## Abstract

Obesity increases insulin resistance and disregulation of glucose homeostasis. This study investigated low glycemic index starch (LGIS)/diacylglycerol (DAG) diet on plasma insulin and circulating incretin hormones during canine weight loss. Obese Beagle dogs were fed one of four starch/oil combination diets (LGIS/DAG; LGIS/triacylglycerol (TAG); high glycemic index starch (HGIS)/DAG; and HGIS/TAG) for 9 weeks during the weight loss period. At weeks 1 and 8, fasting plasma insulin, glucose, nonesterified fatty acid (NEFA), glucose-dependent insulinotropic polypeptide (GIP), and glucagon-like peptide-1 (GLP-1) were determined. Weight loss did not affect fasting insulin, glucose, and NEFA, but fasting GIP increased and GLP-1 decreased. LGIS affected postprandial insulin at both times and glucose was similar to insulin, except 60 min postprandially with DAG at week 8. NEFA lowering was less with the LGIS diets initially but not thereafter. At 60 min postprandially on week 8, GIP was significantly elevated by DAG, while GLP-1 was increased only with the HD diet. LGIS suppressed insulin and glucose responses up to 180 min postprandially at both sample times. DAG increased incretin hormones as did the DAG/HGIS combination but only at week 8. This latter finding appeared to be related to the glucose response but not to insulin at 60 min.

## 1. Introduction

Obesity is a common nutritional disorder both in human and companion animals. The incidence of obesity in humans and dogs is considered to be 33.2% in the USA [1] and between 22 and 40% in Western countries [2–5], respectively. Obesity is associated with metabolic abnormalities including the ablation of regular glucose homeostasis and insulin resistance [6, 7]. Weight reduction coupled with exercise has been shown to improve insulin resistance and delayed onset of diabetes in humans [8–10]. In addition, careful choice of dietary nutrients, such as diacylglycerol (DAG) and low glycemic index starch (LGIS), may have the potential to improve such abnormalities.

In order to elucidate possible effects of DAG and LGIS on hyperinsulinemic responses in dogs, we previously investigated the postprandial effects of a single meal containing 20 g of DAG oil and 25 g of either LGIS or high glycemic index starch (HGIS) mixed with 60 g of boiled boneless chicken breast fed to healthy intact female adult Beagles [11]. Results indicated that the LGIS diet groups significantly lowered plasma insulin concentrations during a 6 h postprandial period while maintaining glucose concentrations. The LGIS diets also increased nonesterified fatty acid (NEFA) mobilization in the systemic circulation. Although this preliminary study found the potential to improve insulin sensitivity by starch type, several researchers reported that DAG, specifically the 1,3-DAG isomer, also elicits a positive effect on insulin sensitivity in addition to obesity reduction [12, 13]. It was, therefore, hypothesized that postprandial insulin concentrations would be lowered using a dietary combination of LGIS and DAG during canine weight loss. Specifically, the objective of this study was to evaluate the extent to which postprandial plasma insulin concentrations may be lowered by longer term (i.e., 9 weeks) feeding of DAG when combined with either LGIS or HGIS.

Weight loss is commonly used as one strategy for improving insulin sensitivity. Therefore, weight loss was induced during this study via energy restriction using the above oil- and starch-containing diets. Furthermore, in humans, glucose-dependent insulinotropic polypeptide (GIP) and glucagon-like polypeptide-1 (GLP-1) have been identified as incretin hormones that potentially play a role in the glucose-dependent insulin response [14]. GIP and GLP-1 have been shown to be secreted from K and L cells of the intestinal wall, respectively, within a few minutes after food ingestion [15, 16]. A second hypothesis was that these incretin hormones would be decreased during the early postprandial period along with plasma insulin and glucose concentrations in obese dogs fed LGIS/DAG diet for weight loss. Here the objective was to compare diets containing HGIS and either DAG or TAG under similar weight loss conditions.

## 2. Materials and Methods

### 2.1. Animals

Twelve obese, sexually intact adult female beagles, 2 to 6 yr of age, with body condition scores (BCS) of 8.4 ± 0.1 (SEM) on a 9 point scale and 48.9 ± 3.3% body fat were used (Table 1). Dogs were individually housed in kennels which were 2.4 m long, 2.7 m high, and 1.2 m wide with 12 h light cycles at the Laboratory Animal Research and Resources facility, Texas A&M University, according to the American Physiological Society Guidelines for Animal Research and according to guidelines set forth by Texas A&M University Care and Use Committee.

The dogs were allowed free access to water and exercise during the study. Prior to entering the study, all dogs had complete blood counts and serum biochemistry profiles performed to assure normal clinical status.

### 2.2. Diets and Feeding

Four experimental diets were prepared using a mixture of starch (LGIS versus HGIS) and oil (DAG versus triacylglycerol (TAG)) types: LGIS/DAG (LD diet), LGIS/TAG (LT diet), HGIS/DAG (HD diet), and HGIS/TAG (HT diet). These diets were formulated in our laboratory using a mixture of 430 g/kg of chicken byproduct meal (Tyson Foods), 135 g/kg of DAG or TAG enriched dietary oil (Kao Corporation), and 430 g/kg of LGIS or HGIS to provide the same amount of macronutrients in each diet (crude protein, 33.0%; fat, 23.0%; carbohydrate, 38.7%; crude fiber, <2.0%; Ash, 5.3%). Five g/kg of a vitamin/mineral premix for dogs (Akey Industries) was also added. Gelatinized high amylose corn starch and waxy corn starch were used as the LGIS and HGIS sources, respectively (Nihon Shokuhin Kako). The DAG and TAG oils in combination with the other diet ingredients contained similar fatty acid compositions whose data were shown in an earlier publication [17]. In order to eliminate composition alterations by batch differences, all ingredients except oils were homogenized together using a mixer (Hobart Industries) at Texas A&M University and stored in a dark ambient temperature-controlled storage room in our laboratory before the study started and were used throughout the study. The homogenized ingredients had a powdered texture, to which 2-3 volumes of water (approximately 2500 g/kg homogenized powder diet) and oils were added before feeding. After mixing these homogenized powders with oil and water, all diets had a gruel-like appearance due to the presence of the gelatinized starches.

Prior to entering the weight loss study, obesity had been induced in all dogs. During this induction period, the dogs were fed a high-fat diet containing dry food (Science Diet Adult Original, Hill's Pet Nutrition) and a mixture of canola and soy bean oils (40 g). Pecan shortbread cookies (Keebler Sandies, Kellog Co.) were also added daily to increase calorie intake overall. Once the dogs reached obese body weights based on BCS and body fat%, the pecan shortbread cookies were removed and their obese body weights were maintained for an additional 2 months. These additional months allowed the dogs to establish a more metabolically stable form of obesity. Four weeks prior to the weight loss study, all dogs were fed a diet containing a combination of a 50/50 (v/v) blend of canola and soybean TAG oils, a 50/50 (w/w) mixture of the HGIS and LGIS, chicken byproduct meal, vitamin/mineral premix, and 2-3 volumes of water as an acclimation diet in amounts calculated to maintain their obese body weights (MER, kJ/d = 523 × (obese  body  weight)_kg_^0.75^). This diet provided similar macronutrient and fatty acid compositions as the experimental diets and with a similar texture. During this acclimation period, it was discovered that the dogs only consumed approximately 70% of the amount fed. Therefore, in the study period, the same obese MER amount of the experimental diets was offered to the dogs in order to achieve body weight loss due to negative energy balance. Indeed, it was found that the dogs voluntarily consumed 68 ± 4% (mean ± SEM) of food offered per day overall independent of starch and oil types. This low consumption may have occurred due to lower palatability because no additional palatability enhancer or flavors was added to the diets.

At week 1, the dogs were randomly assigned into 4 groups (*n* = 3/group) according to age, body weight, and BCS to minimize bias and fed one of the experimental diets (LD, LT, HD, or HT) as described above for 9 weeks. The diets were prepared each morning during the acclimation and experimental feeding periods. All food was removed from the kennels 5 h after feeding and weighed. Body weight was monitored weekly. Body fat was measured at weeks 1 and 9 using a body fat analyzer (Kao Corporation). This study utilized a partial cross-over design (total *n* = 6/diet group). Thus, each dog was fed two of the four diets after an appropriate wash out period as described by Nagaoka et al. [18]. Briefly, after the first 9-week regimen (period 1), obesity was reinduced. This process required 10 weeks to achieve the same degree of obesity and was maintained as noted above. Dogs were then again fed the acclimation diet for 4 weeks followed by assignment to a treatment diet for 9 weeks exactly opposite in starch and oil type to the one that they had been fed during period 1 of the study (i.e., if fed LD in period 1, they were then fed HT in period 2).

### 2.3. Blood Samples

At weeks 1 and 8, jugular catheters were placed in order to conduct postprandial blood collections. A preliminary study found that the starch effect was more dynamically changed during the first 3 h postprandial period. Therefore, blood was collected 3 h postprandially in the present study. Feed had been withheld from the dogs overnight prior to time 0 min blood sample collections. Meals for postprandial sample collections were prepared with a mixture of either 8 g TAG or DAG enriched oil, 25 g LGIS or HGIS, and 80 g boiled chicken breast meat for better palatability and rapid consumption. These four meals had similar macronutrient compositions. Because it was critical that the dogs consumed these meals quickly, approximately 30% of the obese, daily MER amount was prepared for this meal (i.e., ca. 1150 kJ). All dogs consumed their meals within 5 min. Blood samples were then collected at 15, 30, 60, 120, and 180 min after the dogs completed the meals. Samples were placed into EDTA-containing tubes for plasma separation by low speed centrifugation. A protease inhibitor (0.6 TIU/mL blood of aprotinin, Sigma-Aldrich) was added to blood samples for insulin analysis to prevent proteolysis prior to centrifugation. For GIP and GLP-1 analyses, 10 *μ*L of dipeptidyl peptidase IV inhibitor (DPP-IV inhibitor, Linco Research) was added per mL of blood in order to avoid degradation of these incretin hormones [19]. All plasma samples were stored frozen at −80°C until the time of analysis.

### 2.4. Analyses

Postprandial plasma samples were analyzed for glucose and nonesterified fatty acids (NEFA) using enzymatic and colorimetric assays. Mercodia Porcine Insulin ELISA (Mercodia AB) was used for insulin analyses according to Bennet et al. and Sato et al. [20, 21]. GIP (Human) EIA Kit (Phoenix Pharmaceuticals) was appropriately validated by spiking with standard GIP and by serial dilution techniques, and used for GIP analysis. GLP-1 (Active) ELISA Kit (Linco Research) was used for GLP-1 analysis [22]. A microplate spectrofluorometer (Gemini EM, Molecular Devices Corporation) and its software (Softmax Pro ver. 5.0, Molecular Devices Corporation) were used to determine GLP-1 concentrations and a kinetic microplate reader (Molecular Devices Corporation) was used for the other parameters.

### 2.5. Statistical Analyses

Data were expressed as means ± SEM and SPSS 15.0 for Windows was used exclusively for the statistical analyses. Repeated measures ANOVA was performed using a general linear model for fasting samples with oil types (DAG versus TAG) and starch types (HGIS versus LGIS) as between-subjects factors, and week as a within-subject factor with blocking periods (periods 1 and 2) in order to avoid confounding any treatment effect due to the two separate study periods employed. When significance was observed in this model, further pairwise comparison analyses were conducted to obtain simple effects at each treatment level or interactions using Bonferroni corrections. For postprandial samples, the data was converted to area under the curve (AUC) and a two-way ANOVA blocking on period model was used. During the study, all dogs lost body weight (*P* < 0.001). Therefore, for those data obtained at week 8, body weight loss% (based on week 1 body weights) was included in the above-mentioned models for determining possible starch, oil, and oil × starch interaction effects independent of body weight. Normality of dependent variables and homogeneity of population variances were analyzed before all tests were conducted. If data was nonnormally distributed, appropriate non parametric tests were performed. Where variances were not homogeneous, data was transformed as log_10_. Differences were considered significant at *P* < 0.05.

## 3. Results

### 3.1. Body Weight and Body Fat

All dogs lost significant amounts of body weight and body fat during the study (Table 1). However, the degree of weight loss was higher in the LGIS diet groups than the HGIS diet groups (*P* = 0.008). The percentage of body fat lost, however, was not altered by starch and oil types.

### 3.2. Plasma Glucose, Insulin, NEFA, GIP, and GLP-1 Responses

Fasting plasma glucose, insulin, and NEFA concentrations were not significantly different by time, starch and oil types, or interactions (Table 2). Similarly, fasting GIP and GLP-1 were not altered by starch and oil types. However, a time effect was observed for both fasting plasma GIP and GLP-1 concentrations between weeks 1 and 8. Fasting GIP concentrations were significantly increased at week 8 versus week 1 (*P* = 0.013), while fasting GLP-1 concentrations were significantly decreased at week 8 versus week 1 (*P* = 0.001).

Varied postprandial plasma responses based on AUC were observed (Table 3). At week 1, a prominent statistically significant starch effect was seen. The LGIS diets resulted in significantly lower AUCs between 0 and 60 min (AUC_insulin, 0–60_) (*P* = 0.004) than the HGIS diet group. This starch effect was also observed at 180 min postprandially (AUC_insulin, 0–180_) (*P* = 0.001). AUC of both insulin and glucose between 0 and 180 min (AUC_insulin, 0–180_, AUC_glucose, 0–180_) were significantly (*P* < 0.001) and nearly significantly (*P* = 0.052) decreased in the LGIS diet groups compared to the HGIS diet groups while that of NEFA (AUC_NEFA, 0–180_) was significantly higher than the LGIS diet groups (*P* = 0.004). No significant starch, oil, or interaction effects of early postprandial GIP and GLP-1 responses (AUC_GIP, 0–60_, AUC_GLP-1, 0–60_) were observed at week 1.

At week 8, a prominent starch effect was consistently found and specifically at 180 min postprandially. Results of AUC_insulin, 0–180_ and AUC_glucose, 0–180_ showed significantly lower responses with the LGIS diets than the HGIS diets (*P* = 0.046 and *P* = 0.041). Similarly, the early postprandial (i.e., between 0 and 60 min) insulin responses were also significantly lower in the LGIS diets than in the HGIS diets (*P* = 0.039). In contrast, the early postprandial glucose (*P* = 0.006) and GIP (*P* = 0.045) responses were significantly increased in the DAG diets compared with the TAG diets. In addition, only the HD diet significantly increased AUC_GLP-1, 0–60_ compared with the other diets (*P* = 0.005). Although the postprandial NEFA response was significantly altered by starch types at week 1, these effects were abolished at week 8.

## 4. Discussion

The aim of the present study was to determine the effects of DAG, LGIS, and combination of DAG and LGIS on postprandial plasma insulin response in adult obese Beagles when fed for a 9-week weight loss period. As expected, the dogs lost body weight during this study. Additionally, the LGIS diet group lost a greater amount of body weight. The rate of weight loss of LGIS and HGIS diet groups was 1.9 ± 0.2% and 1.0 ± 0.4% per week, respectively, the range of which is within normal limits of that generally recommended for weight loss [17, 23]. Body weight loss did not alter fasting plasma glucose, insulin, and NEFA concentrations but a significant time effect was seen during the study resulting in increased fasting plasma GIP and decreased GLP-1. It is unknown whether this increased plasma fasting GIP and decreased GLP-1 is physiologically relevant because these incretin hormone concentrations are typically low during fasting and rapidly increase following food intake [14, 24, 25]. However, it should be noted that these hormones possess several other functions beyond their incretin effects. For example, GLP-1 has an inhibitory effect on gastric emptying and therefore slows glucose absorption [26]. Moreover, GLP-1 reportedly has an effect on satiety and on reducing food intake [27–30]. Further study will be needed to understand the effect of weight loss on fasting incretin hormone concentrations.

The first objective of this study was to evaluate the long-term effect of DAG and LGIS on postprandial insulin response. In agreement with our preliminary single meal DAG/LGIS feeding study [11], the LGIS diet groups resulted in decreased plasma postprandial insulin concentrations at both weeks 1 and 8. Moreover, in the present study, glucose response was also suppressed by the LGIS diets followed by the insulin response. The slower digestion of a starch type such as amylose which was the LGIS source in the present study would have expectedly provided less glucose flux into the circulation, leading to decreased insulin concentrations [31]. The interesting finding observed in the present study was that although HGIS has been reported to increase insulin concentrations in relevant studies, in combination with DAG, its postprandial concentration was suppressed at week 1 compared with the HGIS/TAG combination. Meguro et al. evaluated the DAG effect on insulin response in rats using a high-fat (DAG or TAG oil) and high-sucrose-containing diet. They found that DAG oil decreased plasma postprandial insulin concentrations compared to TAG oil [32]. Several researchers also reported that DAG possesses a suppressive effect on postprandial insulin, possibly resulting in increased insulin sensitivity [12, 33]. In the present study, a novel finding was that DAG suppressed postprandial insulin only in combination with a highly digestible starch type. However, this DAG effect was attenuated after feeding for 8 weeks and with weight loss. Therefore, it appears that the starch effect was a more dominant factor regarding insulin responsiveness and possibly sensitivity than the dietary oil structure. It should also be noted that the composition of the present experimental diets was considerably higher in starch (43 g/100 g) than in oil (13.5 g/100 g). These higher amounts of starch may therefore have been responsible for a more dramatic primary effect compared to oil type when fed over time.

In addition to the predominant effect of starch type on circulating insulin response, NEFA response was similarly affected and specifically at week 1. Less suppression of postprandial NEFA concentrations with LGIS intake may have been the result of increased hormone sensitive lipase (HSL) activity. The reason for this possibility is that ingestion of LGIS resulted in decreased insulin levels which promote HSL activity and lipolysis [34]. Consequently, if glucose flux into cells had been decreased with LGIS as a result of a decreased insulin response, then relatively more tissue lipolysis from storage sites would be favored, resulting in relatively more fatty acid mobilization and less postprandial NEFA depression. Interestingly, these starch effects on NEFA were abolished by week 8. Although the insulin response was still prominent at week 8 due to starch types, the response *per se*, based on AUC, was lower at week 8 versus week 1. These results suggest that dogs show some adaptation to these two starch types when fed for a longer period.

The second objective of this study was to elucidate the relationship among postprandial incretin hormone responses, insulin, and glucose when DAG and LGIS were fed to obese dogs during weight loss. Incretin hormones are likely to be induced within minutes after food ingestion and their half-life in the circulation is 5–7 min for GIP and 1-2 min for GLP-1 [14, 15]. For this reason we investigated the effects of dietary DAG and LGIS on GIP and GLP-1 in the first 60 min postprandial period. At week 1, the postprandial GIP and GLP-1 response was of small magnitude and no starch or oil effect was observed even though starch types markedly affected the early postprandial insulin response (0–60 min). GLP-1 has been reported to be attenuated by obesity [24, 35] while the effect of obesity on GIP is more equivocal [24, 36–39]. These inconsistent reports regarding GIP are, however, likely affected by study design. For example, Verdich et al. found decreased postprandial GIP response after weight loss compared to before weight loss [24]. In that study, the GIP response was measured before weight loss (obese state) and 6 months after weight loss after feeding a low-fat diet. Although all individuals had consumed the same test meal prior to the blood sample collection, the low-fat, high-carbohydrate, and fiber diet fed likely affected the gastrointestinal steady state over the longer term. Creutzfeldt et al. [38] also found an increased postprandial GIP response in obese subjects, however, this finding was not due to obesity but overeating [36, 40]. Taken together, these results suggest that the lower response of GIP and GLP-1 at week 1 likely occurred due to obesity.

Indeed, after weight loss, postprandial GIP and GLP-1 responses were increased approximately 66% and 11%, respectively, and oil and oil × starch interaction effects were observed. Dietary DAG increased the postprandial GIP and GLP-1 responses but the increased GLP-1 response occurred only in combination with HGIS during the early postprandial period. Moreover, this increase of incretin hormones by DAG was observed along with the plasma glucose response, but not insulin. Shimotoyodome et al. reported that 2 mg/g body weight of DAG administered via gastric gavage significantly decreased area under the GIP response curve during the first 60 min postprandial period [41]. In that study, the lowered GIP response was observed when mice were administered a combination of glucose and DAG oil versus TAG oil. In addition, that study found that plasma GIP concentrations were rapidly increased and reached a peak value within the first 15 min postprandially while this same parameter in the present study appeared to continuously increase after 60 min postprandially. It is noteworthy that the diet composition and carbohydrate sources between their study and the present study were distinctly different. Shimotoyodome et al. used fat alone or a 50/50 glucose/fat combination in their experimental diets, while our experimental diets contained 43.0% starch, 19.7% fat, and 30.0% protein. Therefore, varying diet compositions and the combination of several nutrients may have accounted for the differences observed.

In summary, weight loss did not affect postprandial insulin, glucose, and fat mobilization, while it increased GIP and GLP-1 responses. Starch types were a more dominant stimulus for postprandial insulin, glucose, and NEFA responses than oil types. In the early postprandial period, incretin hormones were increased by DAG which appeared to be associated with glucose concentrations. However, after the first 60 min postprandial period, the DAG effect on glucose response was attenuated and starch types became significant at 180 min postprandially. It is unknown whether this DAG effect on GIP and GLP-1 may be attenuated at 180 min postprandially as well. In conclusion, LGIS improved hyperinsulinemia and hyperglycemia during the 8-week feeding period. Furthermore, fat structure may be one component that alters incretin hormone response during the early postprandial period. However, it remains to be determined whether DAG oil alters incretin hormone concentrations over a longer postprandial period.

## Figures and Tables

**Table 1:** Average body weight, body fat, and daily food consumption of experimental diets during the feeding period.

		Diet				*P* value		
		LD	LT	HD	HT	Oil	Starch	Oil by starch
Body Wt, kg	week 1	15.5 ± 1.1	15.0 ± 1.5	14.3 ± 1.2	14.9 ± 0.7	ns	ns	ns
	week 8	13.1 ± 0.8	12.6 ± 1.2	13.2 ± 1.4	14.1 ± 1.1	ns	ns	ns
	Δ	−14.7 ± 2.2	−15.8 ± 1.4	−8.1 ± 3.0	−6.1 ± 3.8	ns	0.008	ns

Body fat, %	week 1	46.4 ± 3.2	43.7 ± 2.1	43.8 ± 2.2	48.9 ± 3.1	ns	ns	ns
	week 8	37.9 ± 3.0	32.1 ± 2.7	36.3 ± 1.7	39.4 ± 4.5	ns	ns	ns
	Δ	−18.4 ± 2.2	−26.3 ± 5.7	−16.2 ± 5.6	−20.5 ± 4.9	ns	ns	ns

Food intake, g		115.0 ± 9.4	111.8 ± 13.8	125.8 ± 21.4	143.1 ± 14.7	ns	ns	ns
Food intake, kJ		2065.4 ± 187.8	1973.5 ± 254.0	2587.9 ± 429.8	2929.2 ± 396.0	ns	0.038	ns

Mean ± SEM, *n* = 6.; ns denotes no statistical difference. Δ denotes % change, (week 1–week 8) × 100. *P* values are for two-way ANOVA with starch and oil as fixed factors. *P* < 0.05 is considered significant.

**Table 2:** Fasting plasma glucose, insulin, NEFA, and incretin hormones during the feeding period.

		Diet					*P* value			
		LD	LT	HD	HT	SEM	Time	Oil	Starch	Oil by starch
Glucose, mmol/L	week 1	5.2	6.0	5.9	6.0	0.2	ns	ns	ns	ns
	week 8	6.0	5.9	5.8	5.6	0.2		ns	ns	ns

Insulin, pmol/L	week 1	13.0	9.1	8.7	20.6	2.9	ns	ns	ns	ns
	week 8	17.0	9.2	8.5	9.5	1.8		ns	ns	ns

NEFA, mmol/L	week 1	0.8	0.8	0.9	1.0	0.1	ns	ns	ns	ns
	week 8	1.0	0.9	1.0	1.2	0.1		ns	ns	ns

GIP, pmol/L	week 1	5.2	8.9	8.7	9.5	1.0	0.013	ns	ns	ns
	week 8	16.1	10.7	12.1	12.1	1.3		ns	ns	ns

GLP-1, pmol/L	week 1	6.7	7.1	6.6	6.8	0.2	0.001	ns	ns	ns
	week 8	5.6	6.4	6.3	6.0	0.2		ns	ns	ns

Mean ± SEM, *n* = 6.; ns denotes no statistical difference. *P* values for oil, starch, and oil × starch are for two-way ANOVA with starch and oil as fixed factors. *P* value for time effect is for repeated measures ANOVA. *P* < 0.05 is considered significant.

**Table 3:** Fasting and postprandial areas under the curves of plasma glucose, insulin, NEFA, and incretin hormones at weeks 1 and 8 determined at 60 and 180 minutes.

			Diet					*P* value		
			LD	LT	HD	HT	SEM	Oil	Starch	Oil by starch
Week 1	Glucose	Fasting, mmol/L	5.2	6.0	5.9	6.0	0.2	ns	ns	ns
		AUC, 60 min	373.7	332.3	355.4	400.0	14.7	ns	ns	ns
		AUC, 180 min	1117.1	1046.8	1130.5	1261.2	42.1	ns	0.052	ns
	Insulin	Fasting, pmol/L	13.0	9.1	8.7	20.6	2.9	ns	ns	ns
		AUC, 60 min	2074.6	1392.2	3521.0	5548.6	593.0	ns	0.004	ns
		AUC 180 min	15469.2	21485.0	16328.6	18047.4	1986.9	ns	<0.001	0.032
	NEFA	Fasting, mmol/L	0.8	0.8	0.9	1.0	0.1	ns	ns	ns
		AUC, 60 min	34.3	30.4	26.0	31.8	1.7	ns	ns	ns
		AUC, 180 min	91.7	91.3	55.4	61.8	5.8	ns	0.004	ns
	GIP	Fasting, pmol/L	5.2	8.9	8.7	9.5	1.0	ns	ns	ns
		AUC, 60 min	545.2	673.3	839.1	982.5	119.4	ns	ns	ns
	GLP-1	Fasting, pmol/L	6.7	7.1	6.6	6.8	0.2	ns	ns	ns
		AUC, 60 min	453.2	502.2	508.9	494.7	13.9	ns	ns	ns

Week 8	Glucose	Fasting, mmol/L	6.0	5.9	5.8	5.6	0.2	ns	ns	ns
		AUC, 60 min	388.0	373.5	438.7	379.5	13.7	0.006	0.029	ns
		AUC, 180 min	1182.2	1140.1	1308.8	1211.5	41.5	ns	0.041	ns
	Insulin	Fasting, pmol/L	17.0	9.2	8.5	9.5	1.8	ns	ns	ns
		AUC, 60 min	2424.4	1746.2	3922.6	3470.6	375.4	ns	0.039	ns
		AUC, 180 min	18906.8	14180.1	14609.8	18047.4	1429.2	ns	0.041	ns
	NEFA	Fasting, mmol/L	1.0	0.9	1.0	1.2	0.1	ns	ns	ns
		AUC, 60 min	41.1	31.7	29.5	38.3	2.6	ns	ns	ns
		AUC, 180 min	105.7	89.2	75.2	73.6	6.6	ns	ns	ns
	GIP	Fasting, pmol/L	16.1	10.7	12.1	12.1	1.3	ns	ns	ns
		AUC, 60 min	1221.0	1047.6	1736.3	1047.6	124.4	0.045	ns	ns
	GLP-1	Fasting, pmol/L	5.6	6.4	6.3	6.0	0.2	ns	ns	ns
		AUC, 60 min	435.5^a^	618.1^a^	670.8^b^	444.2^a^	35.9	ns	ns	0.005

Mean ± SEM, *n* = 6.; ns denotes no statistical difference. *P* values are for two-way ANOVA with starch and oil as fixed factors.

Letters not in common in a row denote significant differences among diets by two-way ANOVA, *P* < 0.05.

## References

[1] Ogden CL, Carroll MD, Curtin LR, McDowell MA, Tabak CJ, Flegal KM (2006). Prevalence of overweight and obesity in the United States, 1999-2004. *Journal of the American Medical Association*.

[2] Mason E (1970). Obesity in pet dogs. *The Veterinary Record*.

[3] Edney AT, Smith PM (1986). Study of obesity in dogs visiting veterinary practices in the United Kingdom. *The Veterinary Record*.

[4] Donoghue S, Khoo L, Glickman LT, Kronfeld DS (1991). Body condition and diet of relatively healthy older dogs. *Journal of Nutrition*.

[5] Glickman LT, Sonnenschein EG, Clickman NW, Donoghue S, Goldschmidt MH (1995). Patterns of diet and obesity in female adult pet dogs. *Veterinary Clinical Nutrition*.

[6] Misra A, Vikram NK (2003). Clinical and pathophysiological consequences of abdominal adiposity and abdominal adipose tissue depots. *Nutrition*.

[7] Wolever TMS (2000). Dietary carbohydrates and insulin action in humans. *British Journal of Nutrition*.

[8] Goodpaster BH, Kelley DE, Wing RR, Meier A, Thaete FL (1999). Effects of weight loss on regional fat distribution and insulin sensitivity in obesity. *Diabetes*.

[9] Dengel DR, Pratley RE, Hagberg JM, Rogus EM, Goldberg AP (1996). Distinct effects of aerobic exercise training and weight loss on glucose homeostasis in obese sedentary men. *Journal of Applied Physiology*.

[10] Pan XR, Li GW, Hu YH (1997). Effects of diet and exercise in preventing NIDDM in people with impaired glucose tolerance: the Da Qing IGT and diabetes study. *Diabetes Care*.

[11] Bauer JE, Nagaoka D, Porterpan B, Bigley K, Umeda T, Otsuji K (2006). Postprandial lipolytic activities, lipids, and carbohydrate metabolism are altered in dogs fed diacylglycerol meals containing high- and low-glycemic-index starches. *Journal of Nutrition*.

[12] Saito S, Hernandez-Ono A, Ginsberg HN (2007). Dietary 1,3-diacylglycerol protects against diet-induced obesity and insulin resistance. *Metabolism: Clinical and Experimental*.

[13] Li D, Xu T, Takase H (2008). Diacylglycerol-induced improvement of whole-body insulin sensitivity in type 2 diabetes mellitus: a long-term randomized, double-blind controlled study. *Clinical Nutrition*.

[14] Gautier JF, Choukem SP, Girard J (2008). Physiology of incretins (GIP and GLP-1) and abnormalities in type 2 diabetes. *Diabetes and Metabolism*.

[15] Hansen L, Deacon CF, Ørskov C, Holst JJ (1999). Glucagon-like peptide-1-(7-36)amide is transformed to glucagon-like peptide-1-(9-36)amide by dipeptidyl peptidase IV in the capillaries supplying the L cells of the porcine intestine. *Endocrinology*.

[16] Mentlein R (1999). Dipeptidyl-peptidase IV (CD26)-role in the inactivation of regulatory peptides. *Regulatory Peptides*.

[17] Mitsuhashi Y, Nagaoka D, Ishioka K (2010). Postprandial lipid-related metabolites are altered in dogs fed dietary diacylglycerol and low glycemic index starch during weight loss. *Journal of Nutrition*.

[18] Nagaoka D, Mitsuhashi Y, Angell R, Bigley KE, Bauer JE (2010). Re-induction of obese body weight occurs more rapidly and at lower caloric intake in beagles. *Journal of Animal Physiology and Animal Nutrition*.

[19] Kieffer TJ, McIntosh CHS, Pederson RA (1995). Degradation of glucose-dependent insulinotropic polypeptide and truncated glucagon-like peptide 1 in vitro and in vivo by dipeptidyl peptidase IV. *Endocrinology*.

[20] Bennet W, Sundberg B, Lundgren T (2000). Damage to porcine islets of Langerhans after exposure to human blood in vitro, or after intraportal transplantation to cynomologus monkeys: protective effects of sCR1 and heparin. *Transplantation*.

[21] Sato A, Wang PC, Ohgawara H (2002). Effect of stimulators such as GLP-1, PACAP, and nicotinamide on glucose-stimulated insulin secretion from porcine pancreatic endocrine cells in long-term culture. *Pancreas*.

[22] Nathan DM, Schreiber E, Fogel H, Mojsov S, Habener JF (1992). Insulinotropic action of glucagonlike peptide-I-(7-37) in diabetic and nondiabetic subjects. *Diabetes Care*.

[23] Burkholder WJ, Bauer JE (1998). Foods and techniques for managing obesity in companion animals. *Journal of the American Veterinary Medical Association*.

[24] Verdich C, Toubro S, Buemann B, Lysgård Madsen J, Juul Holst J, Astrup A (2001). The role of postprandial releases of insulin and incretin hormones in meal-induced satiety—effect of obesity and weight reduction. *International Journal of Obesity*.

[25] Juntunen KS, Niskanen LK, Liukkonen KH, Poutanen KS, Holst JJ, Mykkänen HM (2002). Postprandial glucose, insulin, and incretin responses to grain products in healthy subjects. *American Journal of Clinical Nutrition*.

[26] Nauck MA, Niedereichholz U, Ettler R (1997). Glucagon-like peptide 1 inhibition of gastric emptying outweighs its insulinotropic effects in healthy humans. *American Journal of Physiology*.

[27] van Dijk G, Thiele TE (1999). Glucagon-like peptide-1 (7-36) amide: a central regulator of satiety and interoceptive stress. *Neuropeptides*.

[28] Flint A, Raben A, Astrup A, Holst JJ (1998). Glucagon-like peptide 1 promotes satiety and suppresses energy intake in humans. *Journal of Clinical Investigation*.

[29] Näslund E, Barkeling B, King N (1999). Energy intake and appetite are suppressed by glucagon-like peptide-1 (GLP-1) in obese men. *International Journal of Obesity*.

[30] Zander M, Madsbad S, Madsen JL, Holst JJ (2002). Effect of 6-week course of glucagon-like peptide 1 on glycaemic control, insulin sensitivity, and *β*-cell function in type 2 diabetes: a parallel-group study. *Lancet*.

[31] Pawlak DB, Kushner JA, Ludwig DS (2004). Effects of dietary glycaemic index on adiposity, glucose homoeostasis, and plasma lipids in animals. *Lancet*.

[32] Meguro S, Osaki N, Matsuo N, Tokimitsu I (2006). Effect of diacylglycerol on the development of impaired glucose tolerance in sucrose-fed rats. *Lipids*.

[33] Yanai H, Yoshida H, Tomono Y (2008). Effects of diacylglycerol on glucose, lipid metabolism, and plasma serotonin levels in Lean Japanese. *Obesity*.

[34] Van Harmelen V, Reynisdottir S, Cianflone K (1999). Mechanisms involved in the regulation of free fatty acid release from isolated human fat cells by acylation-stimulating protein and insulin. *Journal of Biological Chemistry*.

[35] Ranganath LR, Beety JM, Morgan LM, Wright JW, Howland R, Marks V (1996). Attenuated GLP-1 secretion in obesity: cause or consequence?. *Gut*.

[36] Jorde R, Amland PF, Burhol PG (1983). GIP and insulin responses to a test meal in healthy and obese subjects. *Scandinavian Journal of Gastroenterology*.

[37] Sarson DL, Kopelman PG, Besterman HS (1983). Disparity between glucose-dependent insulinotropic polypeptide and insulin responses in obese man. *Diabetologia*.

[38] Creutzfeldt W, Ebert R, Willms B (1978). Gastric inhibitory polypeptide (GIP) and insulin in obesity: increased response to stimulation and defective feedback control of serum levels. *Diabetologia*.

[39] Groop PH (1989). The influence of body weight, age and glucose tolerance on the relationship between GIP secretion and beta-cell function in man. *Scandinavian Journal of Clinical and Laboratory Investigation*.

[40] Willms B, Ebert R, Creutzfeldt W (1978). Gastric inhibitory polypeptide (GIP) and insulin in obesity: II. Reversal of increased response to stimulation by starvation or food restriction. *Diabetologia*.

[41] Shimotoyodome A, Fukuoka D, Suzuki J (2009). Coingestion of acylglycerols differentially affects glucose-induced insulin secretion via glucose-dependent insulinotropic polypeptide in C57BL/6J mice. *Endocrinology*.

